# Illinois State Medical Society

**Published:** 1875-05-01

**Authors:** 


### Editorial Department.

### ILLINOIS STATE MEDICAL SOCIETY.

THE next annual meeting of this Society, is to be held in Jacksonville, commencing on the morning of the third Tuesday in May (the 18th inst.). The place of meeting is one of the most attractive in the state, and the committee of arrangements, with their professional brethren in that city, will spare no pains to make the reception and stay of delegates and members, as pleasant and profitable as possible.

The chairmen of the several standing and special committees, from whom reports are expected, are as follows: On Practical Medicine, E. P. Cook, M.D., of Mendota; on Surgery, Edwin Powell, M.D., of Chicago; on Obstetrics, Hiram Nance, M.D., of Kewanee; on Drugs and Medicines, Wm. E. Quine, M.D., of Chicago; on Necrology, E. Ingalls, M.D., of Chicago; on Ophthalmology, E. L. Holmes, M.D., of Chicago; on Otology, S. J. Jones, M.D., of Chicago; on Diseases of Children, J. O. Hamilton, M.D., of Jerseyville; on Diseases of the Respiratory Organs, H. A. Johnson, M.D., of Chicago; on Galvano - Therapeutics, D. Prince, M.D., of Jacksonville; on Diseases of Women, J. T. Pearman, M.D., of Champaign; on Diseases of the Nervous System, J. S. Whitmire, M.D., of Metamora; on Medical Jurisprudence, R. J. Patterson, M.D., of Batavia; on Dermatology, Chas. G. Smith, M.D., of Chicago; and the Annual Address by the President, J. H. Hollister, M.D., of Chicago. This list of names is a sufficient guarantee that there will be an abundance of reports and papers to occupy the attention of the Society for three days at least. We wish the profession in every part of the state could be induced to organize societies and send delegates to the state and national organizations. It would aid much, both in elevating the character, and extending the usefulness of the whole profession.

"Each local Society shall have the privilege of sending to the Society one delegate for every five of its regular resident members, and one for every additional fraction of more than half of this number. The faculty of every regularly constituted medical college or chartered school of medicine, shall have the privilege of sending two delegates. The professional staff of every chartered or municipal hospital, and every other permanently organized medical institution of good standing, shall have the privilege of sending one delegate.

"*The Members by Invitation* shall consist of practitioners of reputable standing from any part of the United States. They shall receive their appointment by invitation of the meeting, after an introduction from any of the members present, or from any of the absent permanent members. They shall hold their connection with the Society until the close of the session at which they were received, and may participate in the discussions without the right of voting.

"*The Permanent Members* shall consist of regular graduates from reputable schools of medicine, who have served in the capacity of delegates, or such other physicians as may be proposed by two members of this Society, reported on favorably by the committee of investigation, and receiving a two-third vote of all the members present."

A circular will be sent to each member from the committee of arrangements, giving all necessary information relative to the programme, and the arrangements made with the different railroads in regard to reduction of fare, etc.

